# *CYP51* alterations in *Alternaria solani* and their effects on DMI sensitivity and fitness

**DOI:** 10.1128/aem.00400-25

**Published:** 2025-08-28

**Authors:** Ana Carolina Schroeder, Mascha Hoffmeister, Alan De Oliveira Silva, Ian Craig, Gerd Stammler, Holger B. Deising

**Affiliations:** 1 BASF SE, Limburgerhof, Germany; 2 Martin Luther University Halle-Wittenberg, Faculty of Natural Sciences III, Institute for Agricultural and Nutritional Sciences, Phytopathology and Plant Protection, Halle, Germany; 3 BASF SE, Ludwigshafen, Germany; The University of Tennessee Knoxville, Knoxville, Tennessee, USA

**Keywords:** early blight, difenoconazole, mefentrifluconazole, sterol demethylation inhibitors, fungicide resistance, protein model, targeted mutagenesis, Southern blot, fitness penalties

## Abstract

*Alternaria solani,* the causal agent of early blight, may dramatically reduce yield in potato production on a global scale. Quinone outside inhibitors (QoIs), succinate dehydrogenase inhibitors (SDHIs), and demethylation inhibitors (DMIs) represent three major fungicide groups that are used to control early blight on potato. Early blight has acquired adaptation to QoIs and SDHIs, making DMI fungicides the prime class of chemistries in early blight control. We investigated the current DMI sensitivity status of field isolates of *A. solani*. The combination of two mutations leading to the amino acid exchanges L143F and G446S was detected in the DMI target gene *CYP51* in some isolates. Both mutations occurred only in combination and were linked to reduced DMI sensitivity. To test for the role of these mutations in DMI adaptation, we generated strains with single L143F or G446S mutations, as well as L143F + G446S double mutations by targeted mutagenesis. Single mutations caused slightly lower DMI sensitivity; this was more pronounced with the mutation combination L143F + G446S. Importantly, *in vivo* infection assays performed in the greenhouse revealed significantly lower competitiveness of the double mutants compared to wild-type isolates, indicating that the L143F + G446S haplotype is connected with lower fitness. However, *CYP51* haplotypes do not differ in spore morphology or quantity. Furthermore, *CYP51* haplotypes generated by targeted mutagenesis are all able to infect tomato plants in the greenhouse.

**IMPORTANCE:**

This study investigates the mutations in the *CYP51* gene of the fungus *Alternaria solani*, which causes early blight. By understanding the effect of these mutations, researchers can better manage fungicide resistance, ensuring effective disease control. This study provides biotechnological methods suitable for investigating single-site mutations and their individual effect on fungicide sensitivity.

## INTRODUCTION

Potatoes are essential crops and belong to the major food crops worldwide, with the Food and Agricultural Organization statistics database (FAOSTAT) (1) indicating a global potato production of 375 million tons in 2022. Potato production is massively challenged by the causal agent of early blight, *Alternaria solani*, which accounts for yield losses of up to 30% (2). The fungus overwinters as conidia or mycelium on infected plant residues in the soil or on infected tubers (3). Airborne conidia cause first infections on lower leaves and become visible as large, brown, and irregularly evolving necrotic spots with concentric rings (4). Currently, no resistant potato variety exists, but sensitivity varies depending on cultivars and senescence (5–7).

Several foliar fungicides, including quinone outside inhibitors (QoIs), succinate dehydrogenase inhibitors (SDHIs), or demethylation inhibitors (DMIs), are approved for the management of early blight (8). However, the initially effective control of *A. solani* by QoIs was compromised when mutants harboring the F129L mutation in the cytochrome b protein occurred (9, 10), which had also been described in other plant pathogens such as *Phakopsora pachyrhizi* (11) or *Pyrenophora teres* (12). For SDHIs, target site mutations in different SDH subunits, e.g., B-H278R/Y, C-N75S, C-H134R, and D-D123E, have been described in *A. solani*, resulting in sensitivity losses to various SDHI fungicides (8, 13–15). Another class of fungicides registered for early blight control are DMIs. DMIs have been used for many years in potato fields, and no DMI adaptation has been reported in *A. solani* (16, 17). This is in marked contrast to many other pathogens, such as *Zymoseptoria tritici*, *Cercospora beticola*, *Venturia inaequalis,* or *Candida albicans,* where a DMI shift over the last decades is well described (18–24). Different mechanisms have been documented in fungal pathogens that contribute to DMI adaptation: (i) alteration of the target site of the fungicide (20, 22), (ii) upregulation of efflux pumps (25, 26), and/or (iii) overexpression of the target site genes (27, 28). Therefore, fungicide resistance, monitoring, identifying, and understanding mechanisms underlying adaptation is important for risk assessment and successful resistance management in *A. solani*. The aims of this work were (i) to monitor DMI sensitivity in *A. solani* in Europe and Australia by field surveys, (ii) to identify *CYP51* target site mutations in adapted isolates, and (iii) to employ targeted mutagenesis to identify mutations responsible for reduction in DMI sensitivity, fitness, and virulence.

## MATERIALS AND METHODS

### Isolation of field isolates and generation of single-spore isolates of *A. solani*

Two hundred sixty-one isolates of *A. solani* were collected from potato field trials in the Netherlands, Sweden, and Germany in 2022 and 2023 (Table S1). Lesions were cut out from infected leaves with typical early blight symptoms, surface sterilized with 20% sodium hypochlorite for 30 s, then washed with tap water for 5 min and placed onto 2% (wt/vol) malt agar plates supplemented with 30 mg/L streptomycin sulfate (AppliChem GmbH, Darmstadt, Germany). Outgrowing mycelium was transferred to new isolation plates. Isolates were therefore single-lesion isolates.

To generate single-spore isolates (*n* = 235) from samples collected in the Netherlands, Germany, and Australia in 2018, 2020, 2021, and 2022 (Table S1), conidia from each sample were suspended in 700 µL deionized water and vortexed for 30 s. Two hundred microliters of the conidial suspension was spread over malt plates, and five single spores were picked with a sterile needle and transferred separately onto one plate. After 2 to 4 days, germinated conidia were transferred to new plates and incubated for 10–14 days. Plates were incubated at 18°C with 12 h photoperiod (RUMED Typ 1301, Laatzen, Germany).

Two DMI-sensitive wild-type (WT) isolates from Germany, which had been isolated in 1977 and 2022, were used as reference isolates in sensitivity tests.

### Sequencing and *CYP51* haplotype identification

Genomic DNA was extracted from mycelia of 10- to 14-day-old agar plates using the NucleoSpin Plant II Kit (Macherey-Nagel GmbH & Co. KG, Dueren, Germany), according to the manufacturer’s instructions.

PCR primer KES 2539 and KES 2592 (Table S2) were designed to amplify a 1,702 bp *CYP51* and a 1,604 bp coding sequence, based on the whole-genome sequence of the *A. solani* reference strain HWC-168-2012p (GenBank accession: JRWV01000112.1). Sequences of all primers used in this paper are given in Table S2. The 25 µL PCR containing 10 pmol/µL of each primer and 10 ng–50 ng genomic DNA was performed with Phusion Hot Start High-Fidelity DNA polymerase master mix (Finnzymes OV, Espoo, Finland). Amplification parameters were as follows: initial denaturation at 98°C for 2 min; 35 cycles of 98°C for 10 s, 65°C for 20 s, 72°C for 45 s; and final extension of 3 min at 72°C. Amplicons were separated on a 1% agarose gel in 1× Tris-acetate-EDTA (TAE) buffer and stained with pegGREEN DNA/RNA dye (VWR International GmbH, Darmstadt, Germany). PCR products were purified with the NucleoSpin Gel and PCR Clean-up Kit (Macherey-Nagel GmbH & Co. KG) and sequenced at Seqlab-Microsynth (Göttingen, Germany) using primers specified above. Sequences were aligned with a complete *CYP51* WT cDNA sequence with Geneious Prime version 2022.1.1 software (Biomatters, Ltd., Auckland, New Zealand).

### Multiple *CYP51* alignment of different phytopathogenic fungi

Amino acid (aa) sequences of *CYP51* of *A. solani*, *V. inaequalis*, *C. beticola*, *Monilinia fructicola,* and *Pyrenopeziza brassicae* were aligned to visualize the homology of the detected mutations within the *CYP51* sequence. The *CYP51* aa sequences of *V. inaequalis* and *C. beticola* were obtained from strains isolated at BASF SE. The sequence of *M. fructicola* was acquired from the NCBI database (GenBank accession: KY542036), and the sequence of *P. brassicae* was kindly provided by Diana E. Bucur (Teagasc Crop Science Department, Oak Park, Carlow, IE). Alignments with reported sequences (22, 23, 29, 30) were performed using the software Geneious Prime version 2022.1.1 Clustal Omega 1.2.2 (Biomatters, Auckland, New Zealand).

### Homology modeling of *A. solani* Cyp51

To indicate the position of mutations in the *A. solani* Cyp51 protein and to investigate how mutations affect azole binding to the protein, a protein model of Cyp51 was created. As no crystal structure of the *A. solani* Cyp51 was available, Cyp51 of *Aspergillus fumigatus* (PDB ID: 6CR2) (64% sequence identity with *A. solani* Cyp51) was used as template. Homology modeling was done using the software Molecular Operating Environment (version 2020-09, Chemical Computing Group ULC, Montreal, Canada) with standard settings. To aid visualization of the binding site, the pharmaceutical DMI analog VNI [N-1-(2,4-dichlorophenyl) 2-(1H-imidazol-1-yl)−4-(5-phenyl-1,3,4-oxadiazol-2-yl)benzamide] bound to *A. fumigatus* Cyp51 (31) was used to visualize binding of *A. solani* WT and mutant Cyp51 proteins to agricultural DMIs.

### Microtiter plate tests

Sensitivity of all isolates to mefentrifluconazole and difenoconazole was tested in 96-well plates. Spores were suspended in 5 mL–8 mL double-concentrated (d.c.) yeast bacto acetate medium (YBA) per agar plate using a Drygalski spatula and filtered through double-layered cheese cloth (Lohmann und Rauscher GmbH & Co. KG, Neuwied, Germany). Spores were counted in a hemocytometer (C-Chip Neubauer Improve, NanoEntek, Seoul, Korea), and their density was adjusted to 10^4^ conidia/mL d.c. YBA.

The fungicides Revysol (100 g mefentrifluconazole/L emulsifiable concentrate (EC), BASF SE, Limburgerhof, Germany) and Score (250 g difenoconazole/L suspension concentrate (SC), Syngenta Crop Protection, Frankfurt am Main, Germany) were used as commercially available formulations. Dilutions were adjusted with deionized, autoclaved water (18.2 MΩ, total organic carbon [TOC] value: 4) to 3, 1, 0.3, 0.1, 0.03, 0.01, and 0.003 mg/L of the active ingredient. Deionized, autoclaved water served as negative control. Microtiter plate tests were performed as described (22).

### Competition studies *in vivo*

*In vivo* competition studies were performed to investigate if the mutations in the *CYP51* gene cause fitness defects. Single-spore isolates included in this study were from a limited region in northern Netherlands to minimize genetic background heterogeneity (Table S1). Five single-spore isolates harbored the double mutation L143F + G446S and five were WT single-spore isolates. Three mixtures of spore suspension were prepared. The first mixture contained spores of all five WT and all five mutated single-spore isolates (wild-type-mutant mixture). The second mixture contained the spores of five WT single-spore isolates (wild-type mixture), and the third contained spores of the five single-spore isolates of double mutants (mutant mixture). The WT strains used were Ms 1035, Ms 1036, Ms 1037, Ms 1038, and Ms 1039. Double mutants with both L143F and G446S exchanges were Ms 1076, Ms 1077, Ms 1080, Ms 1085, and Ms 1088 (Table S1). All single-spore isolates in all mixtures were used at final concentrations with 10^4^ conidia/mL in a final volume of 50 mL of 0.2% malt extract medium. Each mixture was inoculated with an airbrush (0.8 mm nozzle size) onto three tomato plants until a homogenous coverage of the leaves was visible. Inoculated plants were incubated for 4 to 6 days in a greenhouse chamber at 21°C and 80%–90% relative humidity. Infected leaves were placed onto 2% (wt/vol) malt plates containing 30 mg/L streptomycin sulfate for 2 days at 18°C and a 12 h photoperiod. After 2 days, spores were washed off with 0.2% malt extract medium, and the new spore suspension was inoculated onto new tomato plants. This procedure was repeated for five infection cycles. After each cycle, aliquots of each spore suspension were stored at −20°C for subsequent pyrosequencing and quantifying L143F and G446S mutations. This method is suitable for rigorously testing the competitiveness of single-spore isolates. Using wild-type-mutant mixtures allowed quantifying the shift in the ratio of strains over the cycles. Assays using the WT mixture, or the mutant mixture alone, allowed quantifying the virulence of these strains on tomato plants and served as controls. The trial was conducted twice.

### Vegetative growth of *CYP51* haplotypes

To determine vegetative growth, 2% (wt/vol) malt agar plates were amended with difenoconazole in the following concentrations: 0, 0.01, 0.03, 0.1, 0.3, 1, and 3 mg/L for incubation with the same five field isolates harboring mutation L143F + G446S and five single-spore isolates without mutations used in competition studies. Inoculation of agar plates was conducted with 10^5^ spores/mL. Two biological replicates and three technical replicates per haplotype were performed. The radial growth was measured twice per plate in a 90° angle to each other.

### Spore morphology of *CYP51* haplotypes

To identify differences in spore morphology between WT and mutants, spores of the same isolates used in competition studies were harvested by flooding a 2% (wt/vol) malt agar plate with 2% (wt/vol) malt extract medium. Subsequently, the spore suspension was filtered through double-layered cheese cloth to measure length and width of spores under a light microscope. For WT and double mutation L143F + G446S, five biological replicates were included, and for each single-spore isolate, 100 spores were examined.

### Spore quantity of *CYP51* haplotypes

Spores were quantified to observe spore production of same WT and L143F + G446S isolates as used before. Therefore, 2% (wt/vol) malt agar plates were inoculated by placing 10 µL of a spore suspension, which was prepared with 10^5^ spores/mL and 2% (wt/vol) malt extract medium. After 10 days post-inoculation (dpi), spores were washed off with 2% malt extract medium and filtered through double-layered cheese cloth. The number of spores was then counted twice microscopically in a disposable hemocytometer (Neubauer Improved). Five biological replicates and three technical replicates for WT and haplotype L143F + G446S were included, respectively.

### Infection rate of *CYP51* haplotypes

For determination of infection rate and inhibition by DMIs, greenhouse sensitivity tests were conducted. Targeted mutagenesis strains with mutations L143F, G4446S, L143F + G446S, and two strains with ectopic integration were chosen.

Targeted mutation strains were tested in two independent experiments with DMI fungicide application of the solo-formulated product difenoconazole (Score). Infection rate was visually rated in infected leaf area (%). Fungicide was applied 3 days preventively onto tomato plants with 100 g active ingredient (a.i.)/ha. Untreated plants served as control (UTC), as well as mock control plants. Tap water was used for dilution of formulated products. For inoculation, spore suspensions of *A. solani* were prepared in 0.2% (vol/vol) malt extract medium as described for microtiter-plate tests. Inoculated plants were cultivated for 4 days in a greenhouse chamber at 21°C with 80%–90% humidity.

### Pyrosequencing for L143F and G446S

To quantify the proportion of double-mutant strain mixtures, genomic DNA was extracted from spore suspensions as described above. Subsequently, a pyrosequencing assay was developed using the Pyrosequencing Assay Design software (Qiagen GmbH, Hilden, Germany). A PCR reaction with a 5´ biotin-oligonucleotide and an oligonucleotide without biotin was performed with gDNA and DreamTaq Hot Start Master Mix (Thermo Fisher Scientific, Waltham, MA, USA). For the detection of the L143F exchange, primer pair KES 2917 and 2918 (Table S2) was used, and for the G446S exchange, primer pair KES 2920 and 2921 was used (Table S2). All DNA samples were analyzed in duplicate. Pyrosequencing of codon 143 was performed using primer KES 2899 (Table S2), and for codon 446, primer KES 2922 (Table S2) was used. After PCR amplification, pyrosequencing was conducted as described (32).

### Targeted generation of mutants carrying L143F/G446S single or double exchanges

To investigate whether the *CYP51* mutations identified in field isolates of *A. solani* are associated with reduced DMI sensitivity, three replacement constructs mediating the L143F/G446S single or double mutations were transformed into *A. solani* WT protoplasts of *A. solani* WT strain Li-0016. Each construct consisted of three fragments: a coding region of *CYP51* harboring the respective substitutions, a fragment consisting of the *Aspergillus nidulans trpC* terminator (T_trpc_), the *oliC* promoter, the nourseothricin phosphotransferase (*NAT1*) gene from *Streptomyces noursei* (33), and a fragment of the *A. solani CYP51* terminator (Fig. S1). Twenty base pair tails matching the respective tail of the next fragment were added to the internal primers, thus allowing fusion by double-joint PCR (DJ-PCR) (34).

The 2,278-bp fragment containing the *trpC* terminator, the *oliC* promoter, and the *NAT1* gene was amplified from plasmid Ec47 (provided by Alan De Oliveira Silva, Martin-Luther-Universität Halle-Wittenberg, Germany) (Fig. S5), using primers TtrPC-F3.Unitail and UniNoursR3. The 1,068-bp fragment (containing *CYP51* terminator) was amplified from *A. solani* WT (WT 1092) DNA using the primers KES 2655 and 2656 (Table S2). The first fragment differentiates the three independent constructs, which is shown in Fig. S1. To create the construct mediating both the L143F and the G446S exchange, a 1,258-bp fragment was amplified from the genomic DNA of *A. solani* strain Ms 1030 using the primers KES 2653 and 2654 (Table S2). This fragment contained both the C → T mutation at position 476 and the G → A mutation at position 1434. To generate a construct that carried the C → T substitution and mediated the L143F exchange only, a 1,225-bp fragment was amplified from the WT strain, using the primers KES 2661 and 2654 (Table S2). Forward primer KES 2661 annealed over the first mutation area and harbored the C → T substitution. The construct mediating the G446S mutation, carrying only the G → A substitution, was obtained by amplifying a 1,111-bp- fragment from the genomic DNA of mutant Ms 1092 using primers KES 2659 and 2654 (Table S2). The 1,111-bp fragment spanned the region 3´ of the first mutation until the end of the gene. After joining the fragments by DJ-PCR, the three final constructs were amplified by nested PCR using primer KES 2657 and 2658 for the double substitution, KES 2662 and 2658 for the C → T substitution, and KES 2660 and 2658 (Table S2) for the G → A substitution, resulting in cassettes of 4,613 bp, 4,594 bp and 4,452 bp, respectively.

PCR amplification of these fragments was performed with Phusion High Fidelity DNA Polymerase (Thermo Fisher Scientific). After purification (GeneJet PCR Purification Kit, Thermo Fisher Scientific), fragments were ligated into pJET1.2 (Thermo Fisher Scientific) resulting in vectors pJET1.2-cyp51-L143F + G446S-Nours, pJET1.2-cyp51-L143F-Nours, and pJET1.2-cyp51-G446S-Nours. The cassettes were sequenced with the primers pJET1.2F, KES 2660, KES 2736, KES 2737, KES 2658, KES 2895, KES 2893, KES 2894, and pJET1.2R (Table S2) to confirm correct assembly and nucleotide exchanges. Plasmids were cloned into chemically competent DH5α *Escherichia coli* cells (Agilent Technologies, Santa Clara, CA, USA). Cassettes were PCR amplified from the corresponding plasmids and used to transform *A. solani* WT strain Li-0016.

### Protoplast transformation of *A. solani*

Transformation of *A. solani* followed the protocol describing transformation of *Alternaria alternata* (35), with modifications. *A. solani* spores were harvested from 2% (wt/vol) malt agar plates and, instead of miracloth and glass wool, protoplasts were filtered through double-layered cheese cloth. To the resuspended protoplasts, 5,000 ng of the transformation cassette was added and incubated on ice for 30 min. Additionally, 1 mL of 40% (wt/vol) polyethylene glycol 4000 (PEG4000; Carl Roth GmbH & Co. KG, Karlsruhe, Germany) was added in 1 M Tris-HCl, 0.6 M KCl, 50 mM CaCl_2_, pH 8.0, carefully mixed, and incubated for 20 min at room temperature. Then 12 mL of regeneration medium (1 M sucrose, 0.1% [wt/vol] yeast extract, 0.1% [wt/vol] casein hydrolysate [Sigma Aldrich, St. Louis, USA], 0.6% [wt/vol] agar, 45°C) was mixed with the protoplasts and poured onto selection plates containing regeneration medium with 100 µg/mL nourseothricin and 1.5% [wt/vol] agar. After 3 to 5 days at 25°C, growing colonies were transferred to 12-well potato dextrose agar (PDA) plates containing 50 µg/mL nourseothricin. To obtain homokaryotic transformants, single-spore isolates were generated on isolation plates, as described above.

Transformed single-spore isolates were cultivated in 50 mL 2% malt extract medium with nourseothricin as the selection marker for 12–14 days. Genomic DNA was isolated, and transformants were then screened for the presence of a 3,441 bp PCR fragment indicative of the correct integration, using primers KES 2653 and 2737 (Table S2). Transformed strains yielding the 3,441 bp PCR fragment were selected for Southern blot analysis (Fig. S3). The corresponding transformed *A. solani* strains were named AsL143F, AsG446S, and AsL143F + G446S.

### Identification of single-copy integration of transformation cassettes by Southern blot analyses

To identify transformants with single integration of the replacement cassette, 10 µg of genomic DNA of WT and transformants were digested with the restriction endonucleases *Bsp119I* or *Eco147I* (Thermo Fisher Scientific), and Southern blot analyses were performed (36). Chemiluminescence was visualized by ChemiDoc MP Imaging System (BioRad, Hercules, USA).

### Sensitivity analyses of correct transformants

Transformed strains with correct single integration of the L143F, G446S, and L143F + G446S mutations were evaluated for their sensitivity to mefentrifluconazole and difenoconazole in microtiter plates, as described before.

### Statistics

To calculate whether there were statistical differences among the different haplotypes toward mefentrifluconazole and difenoconazole, statistical analyses were performed using GraphPad Prism version 8.0.2 software executing Kruskal-Wallis test for calculating multiple comparisons between median half maximum effective concentration (EC_50_) values of different haplotypes. As the data were not normally distributed as shown by the Shapiro-Wilk test, the Kruskal-Wallis test was used to examine the significant differences between the different haplotypes for difenoconazole and mefentrifluconazole.

## RESULTS

### Isolates in the field show double mutation L143F + G446S on *CYP51* gene

**Fig 1:**
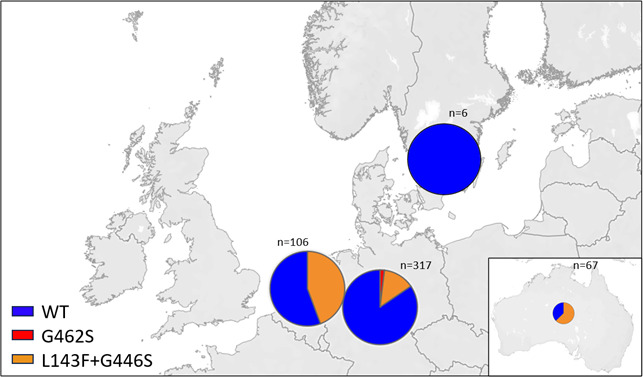
Map with strain collection sites. Collection sites of analyzed *A. solani* single-spore isolates and field isolates are marked in Germany (*n* = 317), Sweden (*n* = 6), Netherlands (*n* = 106), and Australia (*n* = 67). The map was created using Tableau software (Salesforce, Inc., Seattle, WA, USA).

The *CYP51* gene of 496 isolates (235 single-spore isolates and 261 field isolates) from Sweden, the Netherlands, Germany, and Australia was sequenced and compared with the WT sequence. The distribution of detected *CYP51* haplotypes was illustrated using Tableau software (Salesforce Inc., Seattle, USA) (Fig. 1). Single-nucleotide (nt) polymorphisms were identified at three positions (nt 476, nt 1,434, nt 1,482). A total of 161 single-spore isolates and 197 field isolates were without any point mutation within the *CYP51* sequence and belonged to the WT (Table S1). Seventy-two single-spore isolates and 60 field isolates revealed the mutation L143F by a nucleotide substitution from cytosine to thymine at nt position 476. The same single-spore isolates and isolates exhibited a substitution from guanine to adenine at nt 1434, which results in the coding for serine instead of glycine at position 446 (G446S). The mutations L143F and G446S have not been found in a single form but always in combination in the isolates investigated here. Furthermore, four field isolates and two single-spore isolates revealed a single mutation G462S, caused by a nt substitution at position 1482 from guanine to adenine. All field isolates and single-spore isolates were either WT or with a mutated *CYP51* gene, indicating that the field isolates from single lesions represent clones and not populations.

### Multiple *CYP51* alignment of different phytopathogenic fungi

**Fig 2:**
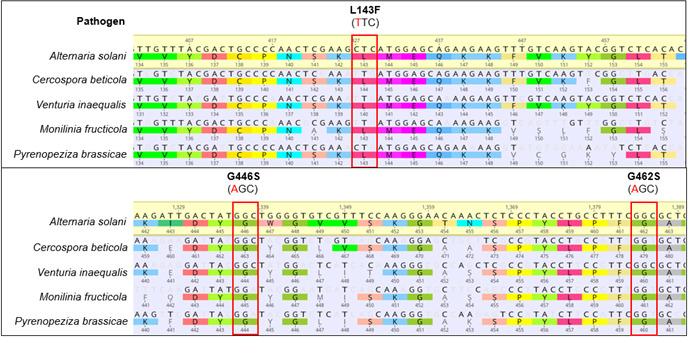
Alignment of nucleotide and amino acid sequences of *A. solani, C. beticola, M. fructicola, P. brassicae,* and *V. inaequalis*. Positions L143, G446, and G462 in *A. solani* WT are marked.

The aa alterations L143F + G446S and G462S detected in this work occurred in highly conserved regions of the protein. A multiple alignment of *CYP51* from the phytopathogenic fungi *C. beticola*, *V. inaequalis*, *M. fructicola,* and *P. brassicae* showed homologous mutations to *A. solani* responsible for reduced sensitivity (Table 1; Fig. 2). Glycine at position 446 in the sequence of *A. solani* is located in a YG region, which is highly conserved in the studied fungi. Due to deletions and insertions in the sequence, the glycine at position 446 in *A. solani* is homologous to G444 in *V. inaequalis*. Leucine at position 143 in *A. solani* is also present in a conserved region in different *CYP51* sequences and is homologous to L140 in *V. inaequalis* and L144 in *C. beticola,* and glycine at position 462 in *A. solani* is homologous to G461 in *M. fructicola* and G460 in *P. brassicae*.

**TABLE 1:** Responsible point mutations in target gene *CYP51* for loss of sensitivity in different fungal pathogens

Species	Mutation in target gene *CYP51*	Literature
*Alternaria solani*	L143F	
*Venturia inaequalis*	L140F	(22)
*Cercospora beticola*	L144F	(23)
*Alternaria solani*	G446S	
*Venturia inaequalis*	G444S	(22)
*Alternaria solani*	G462S	
*Monilinia fructicola*	G461S	(30)
*Pyrenopeziza brassicae*	G460S	(29)

### Homology modeling of *A. solani* Cyp51

**Fig 3:**
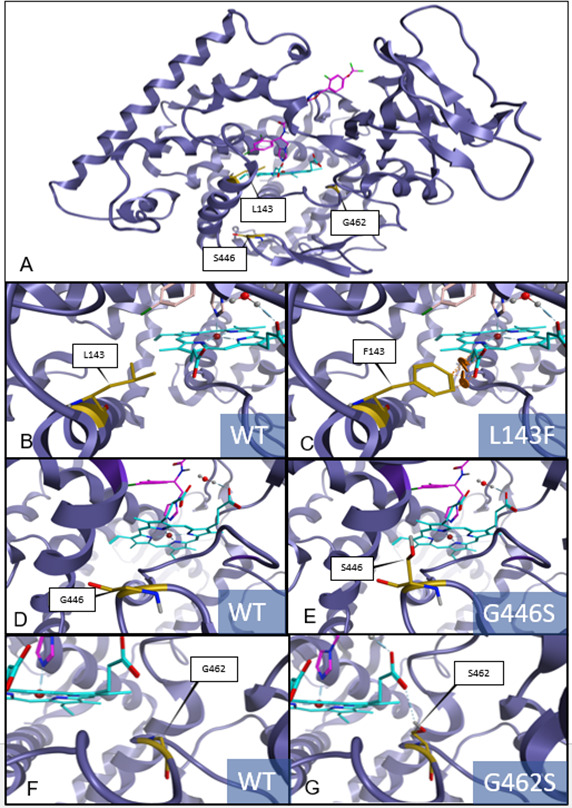
Homology model of the Cyp51 of *A. solani* indicating the location of amino acid alterations L143F, G446S, and G462S. Amino acid position identified as a subject of new alterations is highlighted in yellow, heme group is indicated in light blue with central iron ion marked with a red-brown ball, and the analog fungicide is indicated in pink. WT aa position L143 and mutated aa position F143 (**A, B, C**), WT position G446 and mutated S446 (D, E), and WT G462 and mutated S462 (F, G) are shown in yellow.

The *A. solani* protein model of Cyp51 can be used to locate the position of the detected mutations in relation to the prosthetic heme group of the protein. In Fig. 3A, the prosthetic heme group representing the binding site to azoles is indicated in light blue, the analog DMI in pink, and the newly found mutations in yellow. The position G446 is not in direct interaction with the prosthetic heme group. The binding site surrounding position L143 is shown in a close-up view in Fig. 3B. The aromatic ring of phenylalanine is only a very short distance away from the carboxylate group of the heme (Fig. 3C). This may cause a steric clash that would be energetically unfavorable and is likely to force a change in the position of surrounding heme and protein atoms.

Figure 3D and E illustrate the position of G446 and the altered aa S446. Mutation G446S is not in direct interaction with the prosthetic heme. In Fig. 3F and G, WT position G462 and altered aa S462 are shown to illustrate the distance to the prosthetic heme.

### Sensitivity of different haplotypes using microtiter plate tests

All 235 single-spore isolates and 261 field isolates were analyzed for their sensitivity toward mefentrifluconazole and difenoconazole, and the EC_50_ values were calculated.

**Fig 4:**
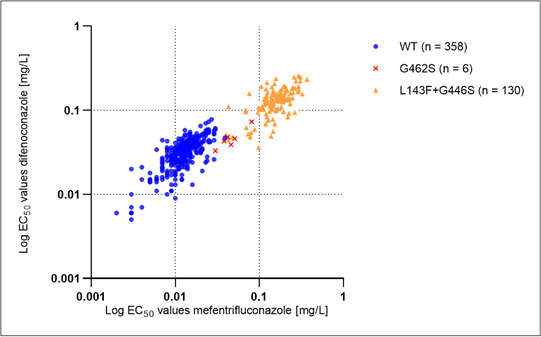
Correlation of EC_50_ values of different *A. solani* haplotypes (WT, G462S, L143F + G446S) for mefentrifluconazole and difenoconazole. To calculate the correlation (*r* = 0.8706), the log-transformed EC_50_ values of all isolates were used. EC_50_ values for difenoconazole and mefentrifluconazole available are plotted in the diagram, and the number of isolates is shown.

Among the WT isolates tested, the EC_50_ values for mefentrifluconazole ranged from <0.002 to 0.039 mg/L with a median of 0.013 mg/L. EC_50_ values for difenoconazole varied from 0.005 to 0.078 mg/L with a median of 0.033 mg/L. When comparing the EC_50_ values of the double-mutant L143F + G446S for mefentrifluconazole and difenoconazole, differences in the sensitivity of the haplotypes were observed (mefentrifluconazole: Kruskal-Wallis, *P* < 0.0001; difenoconazole: Kruskal-Wallis, *P* < 0.0001). EC_50_ values of the double mutants tested varied from 0.040 to 0.367 mg/L (median 0.157 mg/L) for mefentrifluconazole and from 0.036 to 0.256 (median 0.135 mg/L) mg/L for difenoconazole. For both substances, the EC_50_ values of G462S isolates overlapped with the higher EC_50_ WT values. EC_50_ values for difenoconazole were 0.033 and 0.073 mg/L, with a median of 0.045 mg/L, and for mefentrifluconazole 0.030 and 0.081 mg/L, with a median of 0.044 mg/L. The sensitivity to mefentrifluconazole and difenoconazole showed a high correlation with *R*^2^ = 0.75 (Fig. 4).

### Competition studies reveal fitness penalty for isolates with the double mutation

**Fig 5:**
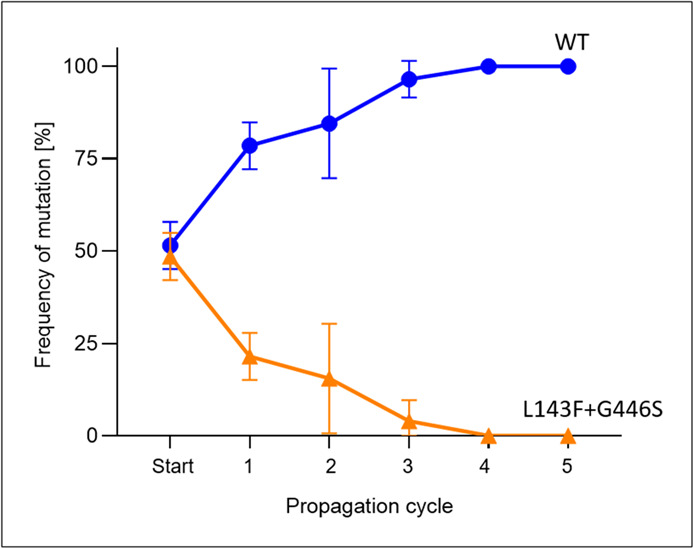
Frequency of mutations in the *CYP51* gene in competition studies of sensitive (WT) and mutated single-spore isolates (L143F + G446S) of *A. solani* during five disease cycles.

To determine the frequency of single-spore isolates which contained L143F + G446S in *CYP51* gene during several infection cycles of the fungus, competition studies in the glasshouse were performed with a mixture of sensitive and adapted *A. solani* single-spore isolates. The initial spore suspension of the wild-type-mutated mixture contained, as intended, around 50% of the L143F and G446S in both trials. A decrease in the frequency of the double-mutated single-spore isolates was observed already after the first infection cycle to a mean of 21%. In the following cycles, the double mutants decreased further and after four cycles to 0% (Fig. 5). After an additional fifth cycle, the frequency remained consistent at 0%. This indicated that single-spore isolates with the double mutant were less fit than the WT when there was no DMI selection pressure. As expected, the mixtures with only WT single-spore isolates were continuously at 0% and the mixtures with only double mutants were at 100% of L143F and G446S.

### *CYP51* haplotypes do not differ in spore morphology or quantity

Spores of five *A. solani* isolates with mutation L143F + G446S (*n* = 500) and five without *CYP51* mutation (*n* = 500) were observed under the light microscope to measure length and width. Furthermore, spore morphology was compared. No differences in shape, septation, or length and width of spores were observed. The length of WT spores varied from 72.7 µm to 351.1 µm with a mean of 157.7 µm, and the length of spores carrying mutation L143F + G446S varied from 91.7 µm to 281.8 µm with a mean of 160.4 µm. The width in WT spores ranged from 12.7 µm to 32.8 µm with a mean of 20.7 µm, whereas the width in spores with mutation L143F + G446S varied from 13.8 µm to 31.2 µm with a mean of 20.8 µm (Fig. S2A and B). The number of spores with and without *CYP51* mutations did not differ significantly. Spores of WT isolates varied from 4 × 10^4^ to 28.5 × 10^4^ with a mean of 10.1 × 10^4^ spores. Spores with *CYP51* mutation L143F + G446S ranged from 1.5 × 10^4^ to 6.5 × 10^4^ with a mean of 4.1 × 10^4^. No significant difference between spore production of WT and L143F + G446S was noticed (Fig. S2C).

### Vegetative growth of WT isolates is more affected by DMIs than that of mutated isolates

**Fig 6:**
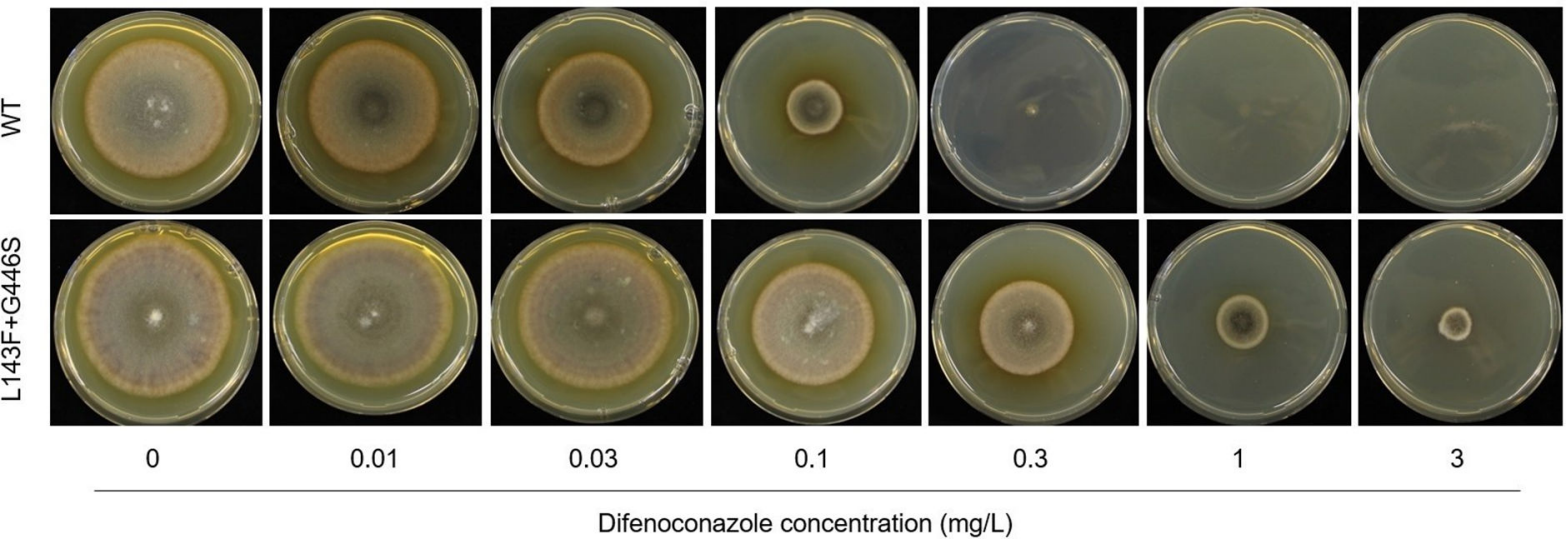
Inhibition of vegetative growth of *CYP51* haplotypes by difenoconazole. Representative sensitive (Ms 1036; WT) and mutated (Ms 1088; double mutation L143F + G446S) single-spore isolate on 2% (wt/vol) malt agar plates amended with 0 mg/L, 0.01 mg/L, 0.03 mg/L, 0.1 mg/L, 0.3 mg/L, 1 mg/L, and 3 mg/L difenoconazole.

Five WT single-spore isolates and five single-spore isolates with mutation L143F + G446S were used to measure the radial growth at 9 dpi on agar plates amended with difenoconazole. Figure 6 illustrates the growth of a representative isolate harboring double mutation L143F + G446S and a WT isolate. The radial growth did not differ on the UTC agar plates between both haplotypes (Table 2). The growth of the WT isolates was fully inhibited at 0.3 mg/L difenoconazole, while the mutated isolates were almost fully controlled at 3 mg/L.

**TABLE 2:** EC_50_ values (in mg/L) of *CYP51* haplotypes tested with difenoconazole in sensitivity agar plate test^*a*^

Isolate	*CYP51* haplotype	Difenoconazole (mg/mL)
Ms 1036	WT	0.055
Ms 1039	WT	0.051
Ms 1077	L143F + G446S	0.421
Ms 1088	L143F + G446S	0.401

^*a*^ EC_50_ values of WT single-spore isolates Ms 1036 and Ms 1039 and single-spore isolates Ms 1077 and Ms 1088 harboring mutation L143F + G446S determined in a sensitivity agar plate test conducted with 0 mg/L, 0.01 mg/L, 0.03 mg/L, 0.1 mg/L, 0.3 mg/L, 1 mg/L, and 3 mg/L difenoconazole.

### Proof of effects of *CYP51* mutations on DMI sensitivity with transformed single-spore isolates

A replacement construct was generated to separate the two mutations L143F and G446S and to transform them into *A. solani* WT. The aim was to investigate the effect of the solo L143F and G446S mutations on the DMI sensitivity and vitality of transformants. In addition, the double mutation was transformed into WT to prove that the mutation combination found is the reason for enhanced EC_50_ values.

In this study, the EC_50_ values of the AsL143F, AsG446S, and AsL143F + G446S single-spore isolates were compared to EC_50_ values of their parental strains. Furthermore, a strain with an ectopic integration was included. A pre-selection using PCR amplification and specific primers (Table S2) was conducted to identify correct integration (Fig. S3). Subsequently, Southern blot analysis was performed to exclude ectopic transformation of the transformation construct (Fig. S1). The results showed enhanced EC_50_ values for AsL143F, AsG446S, and AsL143F + G446S for mefentrifluconazole and difenoconazole when compared to the parental strain. The EC_50_ values of the strain with an ectopic transformation were similar to the parental WT strain (Table 3). Resistance factors (RFs) for difenoconazole and mefentrifluconazole were calculated using the median EC_50_ values of adapted *CYP51* single-spore isolates and divided by the EC_50_ values of the parent isolate. Calculated resistance factors were similar for both compounds. It was observed that resistance factors of AsL143F + G446S were higher than those for AsL143F and AsG446 (Table 3).

**TABLE 3:** Sensitivity of transformed single-spore isolates toward DMIs tested by microtiter plate tests^*a*^

Isolate	*CYP51* mutations	Mefentrifluconazole		Difenoconazole	
		EC_50_ (mg/mL)	RF	EC_50_ (mg/mL)	RF
Parent	WT	0.006		0.016	
L143F (*n* = 6)	L143F	0.019	3	0.039	2
G446S (*n* = 1)	G446S	0.016	3	0.036	2
L143F + G446S (*n* = 4)	L143F + G446S	0.021	4	0.084	5
Ectopic integration	WT	0.006		0.013	

^*a*^ EC_50_ values in mg/L.

### Transformed isolates are able to infect tomato leaves

**Fig 7:**
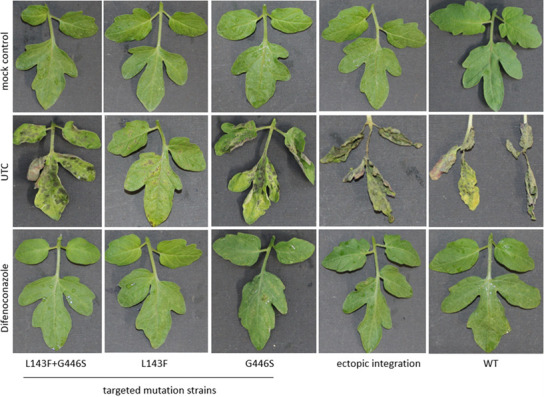
Infection by transformed *A. solani* strains in the greenhouse. Representative transformed *A. solani* strains with different *CYP51* mutations (L143F + G446, L143F, G446S) inoculated on tomato plants in the greenhouse. Parental WT isolate and single-spore isolate with ectopic transformation were included as control.

Greenhouse experiments were conducted by inoculating single-spore isolates with haplotypes L143F + G446S, L143F, G446S, ectopic integration, or WT onto tomato plants treated with the DMI difenoconazole. Figure 7 shows the infection of the different haplotypes on treated and untreated (UTC) tomato leaves. Additionally, a mock control was included. Typical symptoms are visible on the UTC for all haplotypes, and difenoconazole showed a good efficacy with less infection on the leaves. No symptoms were visible on the mock control. On agar plates, vegetative growth of different haplotypes did not differ (Fig. S4).

## DISCUSSION

Selective pressure from chemical and biological agents drives development of diverse resistance mechanisms in fungal plant pathogens. To overcome lethal effects of DMIs, some fungal plant pathogens select aa alterations in the target site of DMI fungicides, overexpress the fungicidal target, and/or enhance efflux and detoxification (37). In many fungal species, the predominant mechanism for a sensitivity shift toward DMIs is mutations in the *CYP51* (20, 22, 30, 38–42). No DMI adaptation has been reported for *A. solani* so far (16, 17), and no *CYP51* mutations in *A. solani* have yet been described. In our study, the DMI sensitivity of the current *A. solani* population was in a narrow range; some single-spore isolates and isolates were shown to have slightly higher EC_50_ values for difenoconazole and mefentrifluconazole. In such single-spore isolates and isolates, mutations in the *CYP51* were detected. These were the mutations L143F and G446S, which occurred only in combination as a double mutation and evolved in conserved domains of the *CYP51*. G462S was also found. Structural analysis of a protein model indicated that the mutations found in *A. solani* evolved near the binding site of the substrate (Fig. 3). At aa position 143, the protein model indicates a steric clash may be possible between the aromatic ring of the phenylalanine mutant and the carboxyl group of the binding site heme (Fig. 3C). The resulting changes in positions of surrounding protein and heme atoms might influence DMI binding affinity. In turn, this might result in reduced sensitivity of *A. solani* strains with the point mutations L143F + G446S and the slight increase in EC_50_ values observed for mefentrifluconazole and difenoconazole compared to the WT. Such protein models have also been developed for other pathogens, e.g., *Z. tritici*, to elucidate how different *CYP51* mutations may affect sensitivity to DMIs (19).

Previous studies have shown that other phytopathogenic fungi developed target site mutations homologous to the mutations found in *A. solani*. The G446S is within a conserved region in fungi (YG). Mutations in this region are well known in different fungi to cause low levels of adaptation, e.g., in *Z. tritici* (42), *Ramularia collo-cygni* (40), or *V. inaequalis* (22). Homologous mutations to L143F have been detected in *C. beticola* (23, 24, 43) and *V. inaequalis* (22) (Fig. 2). The homologous mutation L140F in *V. inaequalis* is so far found only in combination with mutation M141V. Even though the homologous L144F in *C. beticola* can be found alone, it is preferably detected in combination with I309T (and others) (24). Mutations at the homologous aa of G446 in *A. solani* have been detected in *Z. tritici* (20), *R. collo-cygni* (40), and *V. inaequalis* (22) as solo mutations or in combination with other mutations. The presence of the combination of L143F and G446S can have different reasons. One could be that G446S occurred as the first mutation and caused a low adaptation, and then the L143F was added and resulted in a higher adaptation to this. Such additional effects of mutation combinations have been described for several *CYP51* haplotypes in *Z. tritici*. The mutation S524T may serve as an example, since it caused a jump to higher EC_50_ values for various DMI fungicides in several already existing haplotypes without S524T (20). In the human pathogen *C. albicans*, an additional effect of the combination of different mutations in the *CYP51* gene has been observed, demonstrating a stronger impact on resistance to DMIs (21). Another reason for the combination could be that the L143F can only exist in combination with the G446S (or *vice versa*), as it has also been reported for *Z. tritici*: studies with *Z. tritici* indicated that the I381V mutation in *CYP51* can only exist in combination with mutations in the YGY (aa 459–461) region, and that the single I381V mutation without an additional mutation in this region would be lethal (44). I381V was an important step in the evolution of DMI adaptation in *Z. tritici* and is currently present in most *Z. tritici* isolates in Europe (42, 45–47). It was concluded that the occurrence of I381V could not have emerged prior to alterations between Y459 and Y461, which suggested an important role for Y459 to Y461 in the function of the *CYP51* active site (44). G446 in *A. solani* is homologous to G460 in *Z. tritici*. If mutation G446S was required for the evolution of L143F for a functional CYP51, or if L143F and G446S single mutations did not fully impair the CYP51 function, this was more elucidated by the transformation experiments and fitness studies.

*A. solani* WT was transformed with replacement constructs that contained one mutation, the other mutation, and both mutations in combination. The objective of this experiment was to evaluate if one mutation appearing as a single mutation is lethal and to analyze the effect of the solo mutation L143F or G446S on the sensitivity toward DMIs. Additionally, the double mutation was transformed into WT to prove that these mutations are the causal mechanism for the DMI shift. Transformants with the single mutations could be selected and were viable; thus, individual mutations did not fully destroy the *CYP51* functionality, as was the case with the I381V in *Z. tritici* (44). L143F and G446S had increased EC_50_ values for difenoconazole and mefentrifluconazole when compared to the parent. For the double-mutant transformants, the EC_50_ values for both substances were even more increased, and the RF values of the transformed double mutants were close to the sum of the solo transformants. Additionally, the RF values of the transformed double mutants were nearly identical to the RF values calculated for the double-mutated field isolates. These findings proved that the target site mutations L143F and G446S in the *CYP51* gene are responsible for the observed, limited DMI shift in field isolates. Greenhouse experiments on tomato plants showed that transformed *A. solani* isolates are able to infect tomato plants and generate the typical symptoms.

The competitiveness of different haplotypes from the field (WT and L143F + G446S) was tested with single-spore isolates. Ideally, isogenic isolates, including a parent strain and a selected laboratory mutant (preferably selected without mutagenesis), should be used to detect the effect of the mutation. However, this was not feasible within the scope of our studies. Consequently, we selected several isolates from the same year and a limited region to minimize genetic variability. Additionally, these isolates were similar in their resistance to other fungicides to exclude potential fitness penalties caused by other fungicide adaptations.

The mutated strains showed lower competitiveness compared to the WT strains in the glasshouse when they were not exposed to fungicide selection pressure. That indicated that single-spore isolates harboring the mutations L143F + G446S most probably have fitness penalties compared to the WT single-spore isolates and could be less competitive in the field. Similar results have been reported in a study with *A. alternata* where it was demonstrated that fitness of *CYP51* mutants decreased in the absence of fungicides (48). Fitness penalties of isolates with lower DMI sensitivity are also known from *P. pachyrhizi* (11, 49), *M. fructicola* (50), and *C. beticola* (18). Additionally, it was shown that spores of WT and isolates with mutation L143F + G446S did not differ in morphology or sporulation.

Taking the data from the transformation experiments, the fitness studies, and results from other studies on DMI adaptation from other fungal species together, it could be speculated that L143F reduces DMI sensitivity and affects *CYP51* activity negatively but not lethally and that the G446S restores this negative effect partially and has an additive DMI adaptation effect. This would explain the presence of L143F only in combination with G446S, the finding that transformants with single mutations are viable under artificial growth conditions and the fitness penalties of double-mutant field isolates in glasshouse tests.

The ban on multi-site inhibitors, such as dithiocarbamates, in many potato-growing countries, the development of SDHI resistance, and reduced QoI sensitivity resulted in a decreased availability of fungicidal modes of action for efficient early blight control. DMIs are currently important tools for the control of this disease. These studies have shown that DMI adaptation by target site mutations is possible, but that the resistance factors are quite low, and a significant loss of field efficacy is not expected as a result of the currently known target site mutations. Additionally, the *in vivo* tests showed significant fitness costs of the double-mutated isolates. From these findings, it can be expected that resistance management strategies, which include all non-chemical measures for disease reduction and the implementation of fungicides with different modes of action in disease control programs, lead to a sustainable use of DMI fungicides in early blight control. Monitoring studies are further needed to observe the sensitivity toward DMI fungicides and, in the case of changes, to adapt resistance management recommendations.

## SUPPLEMENTAL MATERIAL

Supplemental figures aem.00400-25-s0001.docx Figures S1 to S5.

Table S1 aem.00400-25-s0002.xlsx Characteristics of investigated single spore isolates and field isolates.

Table S2 aem.00400-25-s0003.xlsx Overview of oligonucleotides used in this work.

ASM does not own the copyrights to Supplemental Material that may be linked to, or accessed through, an article. The authors have granted ASM a non-exclusive, world-wide license to publish the Supplemental Material files. Please contact the corresponding author directly for reuse.
