# Supplementary material for: CYP51 alterations in Alternaria solani and their effects on DMI sensitivity and fitness
Source: Appl Environ Microbiol. 2025 Aug 28;91(9):e00400-25. doi: 10.1128/aem.00400-25 (PMC12442347; doi:10.1128/aem.00400-25)
Supplement: Supplemental figures — Figures S1 to S5. [file aem.00400-25-s0001.docx]

Figure S1: Verification of replacement cassettes containing respective substitutions.

Figure S2: *A. solani* spore morphology and sporulation of different *CYP51* haplotypes.

Figure S3: Pre-selection of transformed replacement construct via PCR amplification.

Figure S4: Vegetative growth of amino acid strains generated by target mutated genesis *A. solani*.

Figure S5: Plasmid with primer binding site.


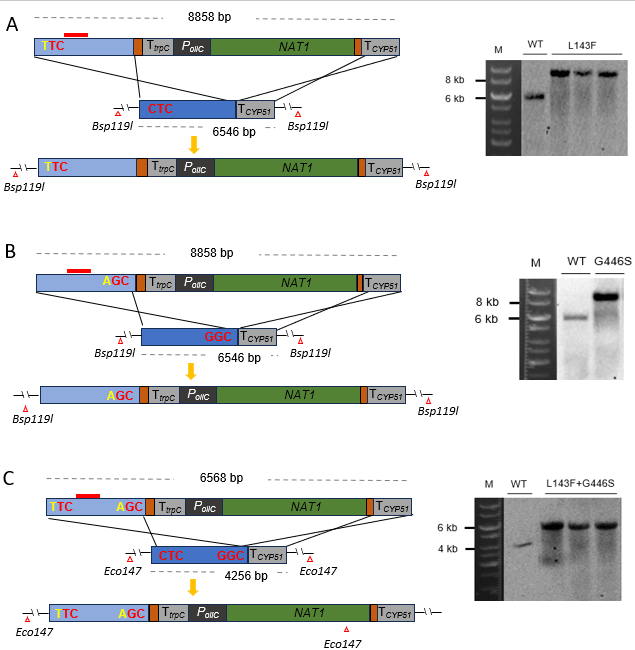


Figure S1: Verification of replacement cassettes containing respective substitutions.

Schematic scheme of transformation construct with mutation L143F (A), G446S (B) and L143F+G446S (C), and southern blots are shown. The brown bars represent the matching tails added to the primers for fragments joining during PCR reaction. Double crossover integration of replacement construct at *CYP51* WT locus is shown. Red bars indicating the probe used for southern blot hybridization, restriction sites of the restriction endonuclease *Bsp119I* and *Eco147* are marked with red arrows. Integrated cassettes with respective substitutions are illustrated.


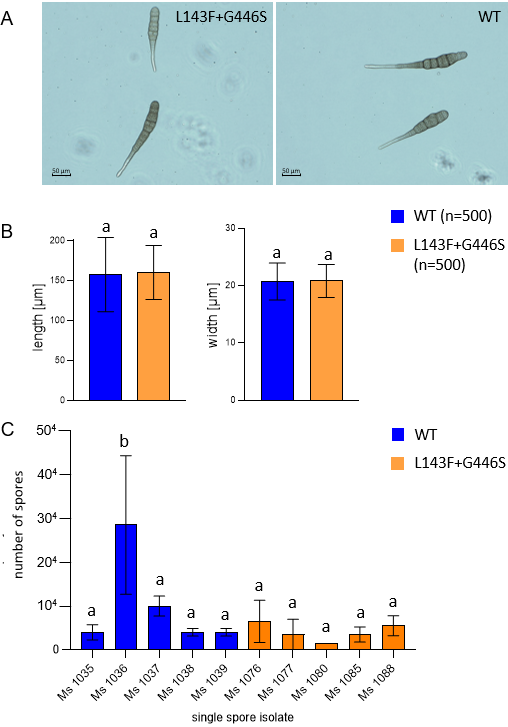


Figure S2: *A. solani* spore morphology and sporulation of different *CYP51* haplotypes.

A: Spore morphology of single spore isolates carrying double mutation L143F+G446S and without mutation in *CYP51,* scale: 50 µm.

B: Spore length and width of five WT single spore isolates (n=500) and five single spore isolates harboring double mutation L143F+G446S (n=500) were measured. The y-scale for length was adjusted to 200 µm and for width to 30 µm. Same letters indicate no significant differences in means at alpha 0.05 according to Mann-Whitney test.

C: Spores of single spore isolates were counted twice under the light microscope and calculated for 3 mL total volume. Ms 1035, 1036, 1037, 1038 and 1039 represent the *CYP51* WT and Ms 1076, 1077, 1080, 1085 and 1088 carry double mutation L143F+G446S.


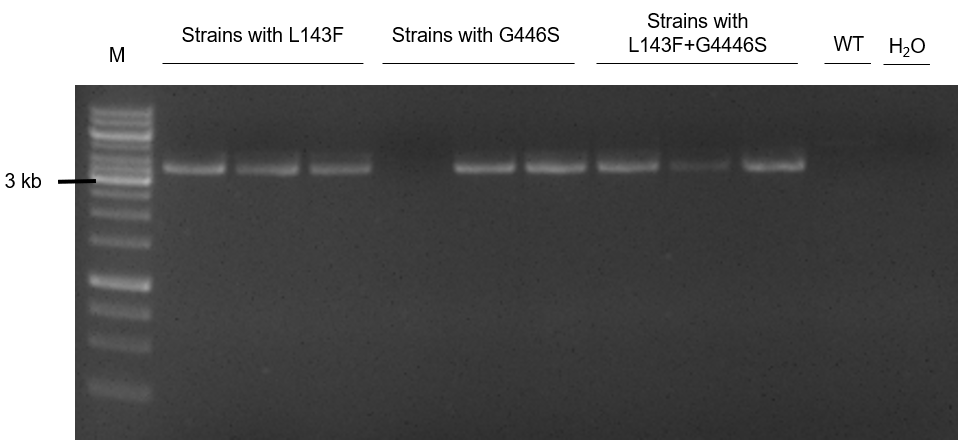


Figure S3: Pre-selection of transformed replacement construct via PCR amplification.

Transformed strains with double mutation L143F+G446S showing a 3440 bp band in agarose gel amplified with primer pair KES 2653 and 2737.


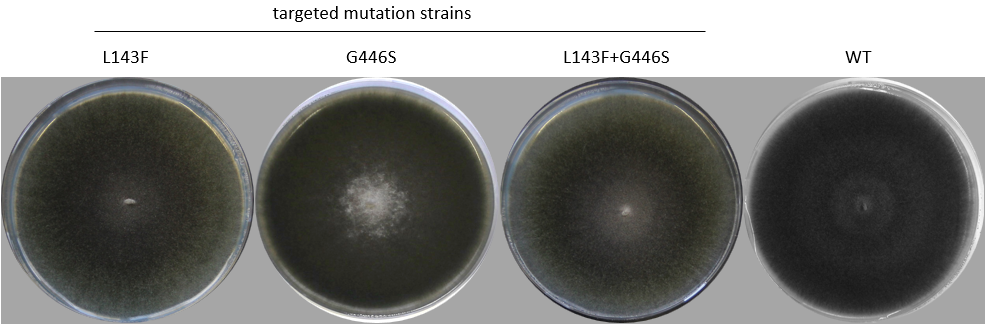


Figure S4: Vegetative growth of amino acid strains generated by target mutated genesis *A. solani*.

Representative transformed *A. solani* strains with different *CYP51* mutations (L143F+G446S, L143F, G446S) and parental WT cultivated on 2% (w/v) malt agar plates (12 dpi).


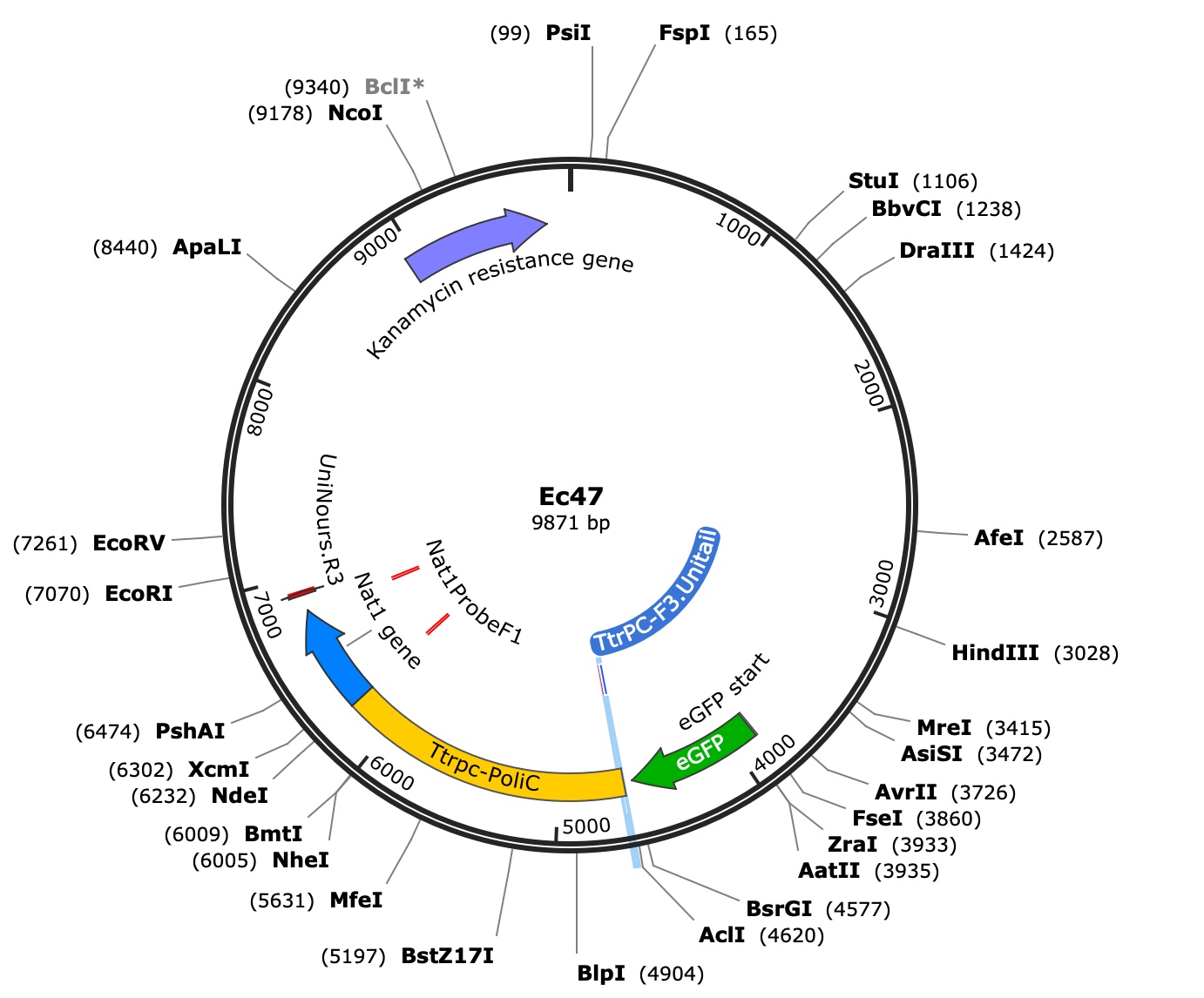


Figure S5: Plasmid with primer binding site.

Plasmid for amplification of the resistance cassette with *Aspergillus nidulans* *trpC* terminator (T_trpc_), the *oliC* promoter (P_oliC_) and the *Nourseothricin Phosphotransferase* (*nat1*) gene from *Streptomyces noursei* is illustrated with primer binding sites for primer pair Ttrpc-F3.Unitail and UniNous.R3.
